# Patient and preceptor attitudes towards teaching medical students in General Practice

**DOI:** 10.1186/1472-6920-13-83

**Published:** 2013-06-07

**Authors:** Otto Pichlhöfer, Hans Tönies, Wolfgang Spiegel, Andree Wilhelm-Mitteräcker, Manfred Maier

**Affiliations:** 1 Department of General Practice and Family Medicine, Medical University Vienna, Kinderspitalgasse 15, Vienna, A-1090, Austria

**Keywords:** Preceptorship, Teaching, Professional ethics, Physician patient relationship, ‘Doctor-patient relationship’, Medical student

## Abstract

**Background:**

Curricula in most western medical universities include teaching in the primary care setting as core elements. This affects GP-teachers, their patients and their interaction. Therefore, it was the aim of this study to assess the influence of the presence of medical students in the teaching practice on the attitudes of both GPs and patients.

**Methods:**

Seventy-four GP-preceptors were invited to answer an online survey. Patients attending consultations with a medical student present completed questionnaires either before the consultation (WR group) or immediately after consultation (AC group).

**Results:**

Fifty- nine preceptors completed the online survey. Physicians showed positive attitudes towards their activities as preceptors: 95% expressed a positive attitude predominantly towards being a role model and to represent the discipline and for 64% remuneration was not important. In 28 practices 508 questionnaires were completed by patients in the WR-group and 346 by the AC-group. Only 12% (WR) and 7.2% (AC) of patients expressed a preference for being seen by the doctor alone. While 16% of doctors rated that confidentiality of the doctor-patient relationship is compromised, only 4.1% (WR) and 1.7% (AC) of patients felt so.

**Conclusion:**

The motivation to be a preceptor is primarily driven by personal and professional values and not by economic incentives. Further, patients have even more positive attitudes than the preceptors towards the presence of students during their consultation. Reservations to teaching students in GP-practices are, therefore, unwarranted.

## Background

For a number of years the need for a more comprehensive and problem-oriented basic medical education relevant for daily clinical practice has been recognized [1]. Since this cannot be taught adequately in the learning environment of a university hospital or a lecture hall, curricula in most western medical universities include teaching in the primary care setting at the community level as a core element. Since the year 2000, the curriculum at the Medical University in Vienna also includes an obligatory attachment to a GP- office. This is an administrative and economic challenge for the academic institution and a challenge of acceptance and managing time constraints for the primary care practices. It particularly affects GP-teachers, their patients and their relationship [2]. However, there is a lack of knowledge regarding their attitudes towards this teaching activity during the consultation [3]. A German study showed that student teaching in primary care practices is well accepted by patients but that it can disturb the relationship between patients and doctors especially if it lacks an informed consent [4]. Open questions are: “How do patients experience the interaction with their GP in the presence of a student?” [5]. “Was there enough time for dialogue and examination?”, “Could privacy be respected if necessary“, “What motivates GPs to act as preceptors?” Some of these questions have been addressed before [4-7]. In the German study referred to, 5% of patients refused to be examined by a student. There is, however, evidence that at least some patients both enjoy taking part in undergraduate teaching and are able to benefit from the process [8]. Since there are no data available from Austria, it was the aim of this study to assess the attitudes of GPs, who are actively involved in teaching, and their patients towards the presence of medical students during the physician- patient interaction.

## Methods

### GPs and patients

The study took place in the offices of General Practitioners in Vienna. All of them were solo practitioners and involved in teaching activities in association with the Department of General Practice and Family Medicine of the Medical University of Vienna for at least 1 year. These teaching activities are voluntary and involve a modest remuneration. Teaching practices are signposted and patients give consent to be seen by a medical student during a consultation on an individual basis. According to the requirements of the Medical University of Vienna, students have to abide to strict confidentiality. Overall 74 GPs were electronically informed about the study and were asked to participate. The GPs who participated in the study did not receive any extra financial compensation for their participation. We did not sample all GPs who would be in principle eligible for a teaching position, since we were exclusively interested in those GPs who already had experience with teaching students in their offices.

Male and female patients older than 14 years of age who consulted the office of a participating GP during the study period were eligible for inclusion. Consecutive patients who agreed to participate were asked to fill in the informed consent. The number of patients who consulted during the study period was recorded.

### Questionnaires

Based on published studies and group discussions among the authors, we developed two questionnaires for patients and one for doctors [4,5,9]. The patient questionnaires were to be administered in the waiting room (WR) and immediately following a consultation (AC). The latter questionnaire contained three additional items concerning the experience during the previous consultation. Both questionnaires had the same questions about patient use of the practice and the interaction with medical students at the practice. The questions about their attitudes to students were similar for both groups with appropriate modifications to the tenses of verbs to fit the timing of the questionnaire. These questionnaires where piloted in one office. The final versions contained 15 and 18 questions respectively.

The questionnaire for doctors contained 21 questions and was to be administered through an online form. The complete sets of items of the questionnaires are presented for doctors (Table 1) and patients (Tables 2 and 3). Because of limited resources and the small population in question a thorough measurement of internal and external validity as well as of the test-retest reliability was not undertaken.

**Table 1:** Doctors questionnaire and responses

	**Never**	**Rarely**	**Frequently**	**Always**
1. In the presence of a student I feel disturbed.	27(46%)	29(49%)	3(5%)	0(0%)
2. In the presence of a student I feel positively motivated.	1(2%)	4(7%)	31(53%)	23(39%)
3. In the presence of a student I feel challenged as a role model.	2(3%)	8(14%)	30(51%)	19(32%)
4. The presence of a student makes additional demands on my time.	7(12%)	22(37%)	26(44%)	4(7%)
5. I have the feeling that the presence of a student enhances my professional status.	8(14%)	7(12%)	23(39%)	21(36%)
6. In the presence of a student there is less dialogue with patients.	30(51%)	28(47%)	1(2%)	0(0%)
7. In the presence of a student there is more dialogue with patients.	3(5%)	18(31%)	29(49%)	9(15%)
8. In the presence of a student there is more small talk.	10(17%)	29(49%)	19(32%)	1(2%)
9. In the presence of a student the confidentiality of the doctor-patient relationship is compromised.	8(14%)	42(71%)	8(14%)	1(2%)
10. The presence of a student facilities the discovery of new information.	2(3%)	39(66%)	17(29%)	1(2%)
11. I feel motivated for this kind of teaching because it suits my professional values.	0(0%)	3(5%)	19(32%)	37(63%)
12. I feel motivated for this kind of teaching because I can present my specialty.	0(0%)	2(3%)	20(34%)	37(63%)
13. I feel motivated for this kind of teaching because I can stay in contact with students.	0(0%)	3(5%)	23(39%)	33(56%)
14. I feel motivated for this kind of teaching because I can stay in contact with university.	3(5%)	12(20%)	27(46%)	17(29%)
15. I feel motivated for this kind of teaching because it is enriching and offers variation.	1(2%)	1(2%)	26(44%)	31(53%)
16. I feel motivated for this kind of teaching because I get a remuneration.	16(27%)	22(37%)	14(24%)	7(12%)
17. I feel motivated for this kind of teaching because I like to teach.	0(0%)	0(0%)	27(46%)	32(54%)
18. I feel motivated for this kind of teaching because it facilitates reflecting on my daily work.	1(2%)	5(8%)	23(39%)	30(51%)

Item response rate is 100%.

**Table 2:** Patients questionnaire: demographic and general items in the waiting room (n = 508) and after consultation (n = 346)

	**WR**	**AC**	***x***^***2***^
*How long have you been a patient in this practice?*			
Less than 3 years	103(21.3%)	81(24.5%)	3.31(2*df*)
3-10 years	128(26.5%)	70(21.2%)	*p* = 0.191
Longer than 10 years	252(52.2%)	179(54.2%)	
*How frequently do you come to this practice?*			
Once a month	258(51.7%)	106(31.5%)	34.47(2*df*)
1-3 times a year	105(21%)	112(33.3%)	*p* < 0.001
Occasionally	136(27.3%)	118(35.1%)	
*Medical students are regular guests in this practice. Was a student present during one of your previous consultations?*			
Yes	392(79.8%)	206(63.4%)	25.154(1*df*)
No	99(20.2%)	119(36.6%)	*p* < 0.001
No answer	18(3.5%)		
*Present at my consultation today was a…*			
Female student		130(37.6%)	
Male student		154(51.4%)	
*The student has listened and has precisely understood my problem.*			
Yes		206(59.5%)	
No answer		140(40.5%)	
*The student has personally questioned/examined me*			
Yes		88(25.4%)	
No answer		258(74.6%)	

Abbreviations: *WR* Waiting room, *AC* After consultation.

**Table 3:** Patients questionnaire: responses to specific statements in the waiting room (n = 508) and after consultation (n = 346)

***If a medical student is present during the consultation I would state as follows:***					
		**Yes**	**No**	**NA**	***x***^**2**^**(1 *df *)**
1. The student interferes (WR) / interfered (AC) with my relationship to my doctor. §	WR	15(3%)	480(94.5%)	13(2.6%)	4.77
	AC	2(0.6%)	334(96.5%)	10(2.9%)	*p* = 0.029*
2. My problem is /was too personal. §	WR	42(8.3%)	444(87.4%)	22(4.3%)	15.45
	AC	6(1.7%)	327(94.5%)	13(3.8%)	*p* < 0.001***
3. I would prefer/have preferred to see my doctor alone. §	WR	61(12%)	416(81.9%)	31(6.1%)	5.16
	AC	25(7.2%)	307(88.7%)	14(9.0%)	*p* = 0.023*
4. I prefer/would have preferred to be examined alone. §	WR	68(13.4%)	419(82.5%)	21(4.1%)	15.45
	AC	17(4.9%)	313(90.5%)	16(4.6%)	*p* < 0.001***
5. The confidentiality seems/seemed compromised. §	WR	21(4.1%)	466(91.7%)	21(4.1%)	3.06
	AC	6(1.7%)	323(93.4%)	17(4.9%)	*p* = 0.08
6. My doctor spends/spent more time	WR	245(48.2%)	201(39.6%)	62(12.2%)	7.22
	AC	131(37.9%)	163(47.1%)	52(15.0%)	*p* = 0.007**
7. My visit to the doctor takes/took longer.	WR	74(14.6%)	399(78.5%)	35(6.9%)	0.589
	AC	57(16.5%)	265(76.6%)	24(6.9%)	*p* = 0.4428
8. Doctors’ explanations to the student help/helped me too.	WR	413(81.3%)	65(12.8%)	30(5.9%)	37.9
	AC	193(55.8%)	93(26.9%)	60(17.3%)	*p* < 0.001***
9. Doctors who teach are medically up to date.	WR	452(89%)	31(6.1%)	25(4.9%)	4.25
	AC	263(76.0%)	32(9.2%)	51(14.7%)	*p* = 0.039*
10. Teaching in practice is helpful for medical students.	WR	483(95.1%)	14(2.8%)	11(2.2%)	2.97
	AC	314(90.8%)	18(5.2%)	14.0(4%)	*p* = 0.085

Abbreviations: *WR* Waiting room, *AC* After consultation, *NA* No answer.

P-Value legend: *** < =0.001; ** = (0.001-0.01); * = (0.01-0.05).

§ Items remaining after optimizing for the best Cronbach’s Alpha.

(Assuming one underlying factor α = 0.750).

### Design of the study

Fifty-nine out of the 74 GP-preceptors, who had agreed to participate, received an e-mail link to an online questionnaire. Two e-mail reminders were also sent out. This part of the study was conducted between May and June 2009.

In the GP offices two groups of patients were asked to fill in a questionnaire. The first group received their questionnaire in the waiting room before or independent of a consultation (WR) and the second group immediately after a consultation where a medical student had been present (AC). For reasons of anonymity patients could deposit the completed questionnaire in a letterbox at the office of the GP or could send it directly to the Department of General Practice in the prepaid envelope.

In preparation for the study, the paper questionnaires and prepaid return envelopes where sent to the participating GP offices. GPs could request more questionnaires if needed. The first stage of the patient study (WR) was conducted between October and November 2009, the second stage (AC) between April and June 2010. The rationale for choosing different groups was to increase response rate and to prevent the bias that would arise through repeated questioning. The study was approved by the Ethics Committee of the Medical University of Vienna (118/2009).

### Data management

The questionnaire for GPs was administered as an online form and the data where downloaded for further analysis. The questionnaires returned from patients were collected and manually entered into SPSS 15 for further analyses.

### Statistical evaluation

The results were analyzed descriptively with SPSS 15. The two groups of patients were compared and several subgroups were analyzed. Data are presented as mean and standard deviation unless indicated otherwise and were compared by Chi^2^ tests with a significance level of p < 0.05 applying Pearson’s correction where appropriate. Assuming one hidden factor that represents the acceptance of students by patients during a consultation we tested the items of the patient questionnaires for internal consistency by Cronbach`s Alpha [10,11].

## Results

After two reminders 59 preceptors completed the online questionnaire (response rate 80%). Their mean age was 53 years (SD 6.5); 55% were female and 45% male.

The most important motivational factors for teaching were represented in items 11–13 (Table 1): “I feel motivated for this kind of teaching because it suits my professional values.” “I feel motivated for this kind of teaching because I can demonstrate my specialty.” “I feel motivated for this kind of teaching because I can stay in contact with students.” For 27% of participants receiving remuneration as a motivational factor was „never important“(Item 16). Sixteen % of doctors felt that “the presence of a student would compromise the patient-doctor relationship” (Table 1, item 9, rated 3 and 4).

For the patient study, 28 practices agreed to participate. The attendance rate varied between 15 and 110 patients per day (mean = 54). However the response rate could not be reliably calculated because the number of patients who declined to fill in the questionnaire was only incompletely recorded. In the waiting room (WR), 508 questionnaires were completed with 33% of respondents females and 57% males (10% gave not answer to this question). In the AC group 346 questionnaires were completed (36% males, 54% females, 10% no answer).

Results from the two patient questionnaires are shown in Tables 2 and 3. Table 2 shows answers to general questions asked in both the WR and AC groups. There was no significant difference between the groups regarding gender distribution and duration of practice attachment (data not shown). Patients in the WR group attended more frequently (p < 0,001) than patients in the AC group. In the WR group 20.2% and in the AC group 36.6% of patients had previously no experience with a student present during their consultation.

Table 3 shows patients responses to more specific statements in the questionnaire. Patients in both groups stated that students did not interfere with the doctor-patient relationship (WR 94% AC 96%, item1). Only 4.1% (WR) and 1.7% (AC) of patients felt that the confidentiality of the doctor-patient relationship had been compromised due to the presence of a student (Table 3, item 5). Whether or not patients had met a student in a previous consultation did not significantly influence the distribution of their answers (data not shown).

In the AC group of the study several subgroups were analyzed. An age over 60 years (data not shown) and longer practice attachment (Table 4) correlated with an affirmation to the question: “My doctor spent more time.” Patients who stated that the doctor spent more time for the consultation also said that his/her explanations to the student helped them too (p = 0.001; data not shown). Of patients who had personally been examined by a student, 26% felt that their consultation lasted longer, while 73% did not think so (data not shown). The mere presence of a student seemed to prolong the consultation for 18% of patients (data not shown).

**Table 4:** Subgroups of AC patients attending the practice 3 to 10 years and over

***My doctor spends more time***		
	**Patient since 3–10 years**	**Patients over 10 years**
Yes	19(27.1%)	76(42.5%)
NO	40(57.1%)	73(40.8%)
NO answer	11(15.7%)	30(16.8%)
Sum	70(100%)	179(100%)
Pearson’s *x*^2^ = 6.095(2*df*); *p* = 0.047		

Further subgroups were analyzed with regard to the items in Table 3. We found no significant differences between genders, whether a student had previously been present during a consultation and between patients attending the practice up to 10 years or over 10 years except for item 6 (Table 3; as shown in Table 4).

When tested for internal consistency of the items in the patient questionnaires by Cronbach`s Alpha, we were left with five items that seem to represent the core message of our study (Alpha 0.77). They are marked by § in Table 3.

## Discussion

This is the first study from Austria about attitudes and experiences of both patients and doctors in GP-teaching practices. The results show that the motivation to be a preceptor is primarily driven by personal and professional values rather than by economic incentives. Further, patients have an even more positive attitude towards the presence of students during their consultation than the preceptors. Therefore, the possible concern of compromising the patient-doctor relationship in a teaching practice is unwarranted.

Doctors (Table 1) felt positively challenged as role models, almost never felt disturbed and were positively motivated by a student’s presence. Their positive attitude was related to professional values, the desire to act as teachers and to stay in contact with students. To receive remuneration was least important. However, since the preceptors in fact receive a reasonable remuneration, their response to this question might be biased. We consider an appropriate remuneration as important because it would demonstrate that the Medical University appreciates and respects the contribution of the preceptors to the teaching program; this in turn would facilitate the recruitment of more teaching practices and their long term commitment to teaching competence. On the other hand, former studies also have shown that the personal motivation for clinical teaching was considered more important than administrative and financial matters [9]. As can also be seen in Table 1, the presence of a student requires additional time of the preceptor. Similar results have been reported in other studies [12-15]. For example, time at work increased by 52 minutes per day and between 2 and 20 minutes per patient.

More than half of patients of both groups had been attending in the respective practices for more than 10 years (Table 2). However, patients of the WR-group consulted significantly more often. This is most likely due to the fact that this group consists of both patients to be personally seen by the GP and those with chronic conditions who require repeat prescriptions only from the administrative personnel. Additionally, some 20% to 36% had previously no experience with a student present during their consultation. This reflects the rather short implementation period of obligatory teaching activities in General Practice in Austria.

Table 3 shows the response of patients to specific statements. Significantly fewer patients in the AC-group agreed with “I would prefer to be examined alone”, “to see my doctor alone” and “my problem is too personal”. Although we were not able to compare the change of opinion in individual patients, nevertheless it appears that the experience of patients during the consultation in the presence of a student might have influenced their attitude. A similar observation has been made by Cooke et al. 1996 [16]. Further, less than 5% of patients in both groups were concerned about compromised confidentiality (Table 3, item 5). In contrast, doctors were more concerned about confidentiality than patients (Table 1, item 9). Our results are in agreement with two other studies [17,18]. More patients in the WR group than in the AC group estimated that the presence of a student would make the consultation last longer (Table 3, items 6 and 7).

As shown in Table 4, significantly more patients who attended the practice for more than 10 years stated that the doctor spent more time. Being personally examined or questioned by students rarely seemed to need additional time (subgroup analysis, data not shown). On the other hand, patients welcomed explanations of the doctor to the student because these helped them too (Table 3, item 8); this was especially seen in older patients (data not shown). These results seem to indicate that a deviation from the usual consultation style in the presence of a student may be more prominently experienced by well known patients.

Since our questionnaire contained a total of 10 specific questions it seems reasonable to identify those of highest relevance. Using Cronbach alpha questions 1–5 turned out to be of utmost importance (Table 3). This confirms the priority of privacy for patients. Intimacy ranked high in other studies too. A study of 4 practices in Germany found that 81% of patients accepted that a student was present. However, 9% declared that they concealed information then [4]. Up to 10% of responders questioned by O´Flynn left the consultation without saying what they wanted to say and 30% found it more difficult to talk about personal matters [19]. Monnickendam reported that 3.2% of the participants objected to the presence of a student during the consultation; 15% would insist on advance notification about the presence of a student, and another 13.9% would request it. Four percent stated that the presence of students had a negative influence on the physical examination and history taking and 33% would refuse to be examined by a student without the doctor´s presence [20]. In a study by Cooke only 3% of respondents expressed negative feelings about having a medical student present whereas 51% felt positively [16]. Overall, the percentage of patients who object to a teaching situation during their consultation is surprisingly small. Our study confirms a similar positive attitude of patients for Austria (Table 3). Nevertheless, their concern or objection must not be neglected.

There are several strengths in our study. For the first time both doctors and patients have been studied in Austria using similar questionnaires which enabled us to make a comparative assessment of doctors’ and patients’ opinions and observations. The sample size is large enough to analyze subgroups such as gender or age of patients or their length of practice attachment. Further, the response rate among doctors was high (80%). Finally, the assessment took place at a rather early stage of implementation of community teaching at our Medical University. This should enable curricular adjustments based on research evidence.

Weaknesses include limited anonymity for the preceptors. However, since the remuneration played a minor part in the overall motivation we did not anticipate a relevant selection bias because of that reason. Further, patients had to sign a lengthy 5-page informed consent form to be included in the study. This might have demotivated some patients to participate. Secondly, the 2 study arms (WR and AC groups) consisted of different respondents with a possible but unlikely overlap. Thirdly, our self designed questionnaire was not thoroughly validated before the study. Patients had to answer the questionnaire while still present in the GP office which might have introduced a response bias in favor of the processes that happen at the office. Further, although the missingness in the item response was only 4% the actual response rate cannot be calculated.

## Conclusions

In conclusion, patients in Austria show predominantly a positive attitude towards a teaching situation during their consultation. We hypothesize that countries with a similar status of academic General Practice can expect an analogous reality. Preceptors and their staff should be skilled to manage situations which require strict confidentiality of personal problems or which require to see the patient alone. Based on our results, however, we believe that reservations of some GPs to participate in teaching students in their practices are unwarranted [21].

Ethical approval was obtained from the Ethics Committee of the Medical University of Vienna (Nr.: 118/2009).

## Abbreviations

GP: General practice; WR: Waiting room (Group of patients that was questioned in the waiting room before or independent of a following consultation); AC: After consultation (Group of patients that was questioned after a consultation in the presence of a student).

## Competing interests

The authors declare no conflicting interests.

## Authors’ contributions

MM had the original idea for the study and supervised the preparation of the manuscript. All cited authors where involved in the planning of the study and in drafting the questionnaires that were used for the study. HT conducted the majority of the data acquisition. OP was mainly responsible for drafting the manuscript. All authors read and approved the final manuscript.

## Pre-publication history

The pre-publication history for this paper can be accessed here:

http://www.biomedcentral.com/1472-6920/13/83/prepub

## Research support

No external financial support, grants or equipment have been obtained for this study.
